# Association between serum alkaline phosphatase and primary resistance to erythropoiesis stimulating agents in chronic kidney disease: a secondary analysis of the HERO trial

**DOI:** 10.1186/s40697-015-0066-5

**Published:** 2015-08-18

**Authors:** Sunil V. Badve, Lei Zhang, Jeff S. Coombes, Elaine M. Pascoe, Alan Cass, Philip Clarke, Paolo Ferrari, Stephen P. McDonald, Alicia T. Morrish, Eugenie Pedagogos, Vlado Perkovic, Donna Reidlinger, Anish Scaria, Rowan Walker, Liza A. Vergara, Carmel M. Hawley, David W. Johnson, on behalf of the HERO Study Collaborative Group

**Affiliations:** Australasian Kidney Trials Network, University of Queensland, Brisbane, Australia; Department of Nephrology, Princess Alexandra Hospital, Brisbane, Australia; Department of Nephrology, Guangdong Provincial Hospital of Chinese Medicine, Guangzhou, China; School of Human Movement Studies, University of Queensland, Brisbane, Australia; Menzies School of Health Research, Darwin, Australia; Centre for Health Policy, Programs & Economics, University of Melbourne, Melbourne, Australia; Department of Nephrology, Prince of Wales Hospital, Sydney, Australia; Department of Nephrology and Transplantation Services, University of Adelaide at Central Northern Adelaide Renal and Transplantation Services, Adelaide, Australia; Department of Nephrology, Royal Melbourne Hospital, Melbourne, Australia; The George Institute for Global Health, Sydney, Australia; Department of Renal Medicine, The Alfred Hospital, Melbourne, Australia; Department of Nephrology, Level 2, ARTS Building, Princess Alexandra Hospital, Ipswich Road, Woolloongabba, Queensland 4102 Australia

**Keywords:** Erythropoiesis stimulating agents, Anemia, Chronic kidney disease, Alkaline phosphatase, Biological marker, Risk factors, Randomized controlled trial

## Abstract

**Background:**

Erythropoiesis stimulating agent (ESA)-resistant anemia is common in chronic kidney disease (CKD).

**Objectives:**

To evaluate the determinants of severity of ESA resistance in patients with CKD and primary ESA-resistance.

**Design:**

Secondary analysis of a randomized controlled trial (the Handling Erythropoietin Resistance with Oxpentifylline, HERO)

**Setting and patients:**

53 adult patients with CKD stage 4 or 5 and primary ESA-resistant anemia (hemoglobin ≤120 g/L, ESA resistance index [ERI] ≥1.0 IU/kg/week/gHb for erythropoietin or ≥0.005 μg/kg/week/gHb for darbepoeitin, no cause for ESA-resistance identified).

**Measurements:**

Iron studies, parathyroid hormone, albumin, liver enzymes, phosphate or markers of oxidative stress and inflammation.

**Methods:**

Participants were divided into tertiles of ERI. Multinomial logistic regression was used to analyse the determinants of ERI tertiles.

**Results:**

All patients, except one, were receiving dialysis for end-stage kidney disease. The mean ± SD ERI values in the low (*n* = 18), medium (*n* = 18) and high (*n* = 17) ERI tertiles were 1.4 ± 0.3, 2.3 ± 0.2 and 3.5 ± 0.8 IU/kg/week/gHb, respectively (*P* < 0.001). There were no significant differences observed in age, gender, ethnicity, cause of kidney disease, diabetes, iron studies, parathyroid hormone, albumin, liver enzymes, phosphate or markers of oxidative stress and inflammation between the ERI tertiles. The median [inter-quartile range] serum alkaline phosphatase concentrations in the low, medium and high ERI tertiles were 89 [64,121], 99 [76,134 and 148 [87,175] U/L, respectively (*P* = 0.054). There was a weak but statistically significant association between ERI and serum alkaline phosphatase (R^2^ = 0.06, *P* = 0.03). Using multinomial logistic regression, the risk of being in the high ERI tertile relative to the low ERI tertile increased with increasing serum alkaline phosphatase levels (*P* = 0.02). No other variables were significantly associated with ERI.

**Limitations:**

Small sample size; bone-specific alkaline phosphatase, other markers of bone turnover and bone biopsies not evaluated.

**Conclusions:**

Serum alkaline phosphatase was associated with severity of ESA resistance in ESA-resistant patients with CKD. Large prospective studies are required to confirm this association. (Trial registration: Australian New Zealand Clinical Trials Registry 12608000199314)

## Résumé

**Contexte:**

L’anémie résistante à l’agent stimulant l’érythropoïèse (ASE) est courante chez les patients atteints d’insuffisance rénale chronique (IRC).

**Objectifs:**

Évaluer les déterminants de la gravité de la résistance à l’ASE chez les patients atteints d’IRC et de la résistance primaire à l’ASE.

**Type d’étude:**

Analyse secondaire d’un essai clinique randomisé (le Handling Erythropoietin Resistance with Oxpentifylline, HERO)

**Contexte et participants:**

53 patients adultes atteints d’IRC de stade 4 ou 5, de même que d’anémie résistante à l’ASE (hémoglobine ≤120 g/L, indice de résistance à l’ASE [IRA] ≥1,0 IU/kg/semaine/gHb pour l’érythropoïétine ou ≥0,005 μg/kg/semaine/gHb pour la darbépoétine, aucune cause justifiant la résistance à l’ASE).

**Mesures:**

Mesure des taux de fer, de la parathormone, de l’albumine, de l’enzyme hépatique, du phosphate ou des marqueurs de stress oxydatif et d’inflammation.

**Méthodes:**

Les participants étaient divisés en tertiels d’IRA. La méthode de régression logistique multinominale a été utilisée pour analyser les déterminants des tertiels d’IRA.

**Résultats:**

Tous les patients, sauf un, recevaient une dialyse pour l’IRT. La moyenne ± ÉT des valeurs d’IRA des tertiels inférieur (*n* = 18), intermédiaire (*n* = 18) et supérieur (*n* = 17) étaient respectivement de 1,4 ± 0,3, de 2,3 ± 0,2 et de 3,5 ± 0,8 IU/kg/semaine/gHb (*P* < 0,001). On n’a observé aucune différence importante en fonction de l’âge, du sexe, de l’ethnicité, de la cause de l’insuffisance rénale, du diabète, des taux de fer, de la parathormone, de l’albumine, de l’enzyme hépatique, du phosphate ou des marqueurs de stress oxydatif et d’inflammation entre les tertiels d’IRA. La médiane [écart interquartile] des concentrations de phosphatase alcaline sérique des tertiels d’IRA inférieur, intermédiaire et supérieur étaient respectivement de 89 [64,121], de 99 [76,134] et de 148 [87,175] U/L (*P* = 0,054). Bien que statistiquement significative, la relation était plutôt faible entre l’IRA et la phosphatase alcaline sérique (R^2^ = 0,06; *P* = 0,03). Selon la méthode de régression logistique multinominale, les chances de se retrouver dans le tertiel d’IRA supérieur, par opposition au tertiel d’IRA inférieur, augmentait avec des taux élevés de phosphatase alcaline sérique (P = 0,02). On n’a observé aucune association forte entre une autre variable et l’IRA.

**Conclusion:**

La phosphatase alcaline sérique a été associée à une forte résistance à l’ASE chez les patients résistants à l’ASE atteints d’IRC. Des études prospectives à grande échelle sont nécessaires afin de confirmer cette association. (Enregistrement de l’essai clinique: Australian New Zealand Clinical Trials Registry 12608000199314)

## Short section

### What was known before

Increased serum alkaline phosphatase is associated with decreased responsiveness to erythropoiesis stimulating agents in patients with end-stage kidney disease.

### What this study adds

Serum alkaline phosphatase is associated with severity of resistance to erythropoiesis stimulating agents in patients with chronic kidney disease with no identifiable causes for resistance to erythropoiesis stimulating agents.

## Background

Since the introduction of erythropoiesis stimulating agents (ESA), there have been substantial reductions in the blood transfusion requirements of patients suffering from chronic kidney disease (CKD) [1]. Unfortunately, 7–14 % of all patients with end-stage kidney disease (ESKD) show a suboptimal hematologic response to ESA (Hb concentration <100 g/L) [2–4]. There are several known causes of suboptimal response to ESA. These include: female gender; [5–8] old age; [7] diabetes mellitus; [9] cardiovascular disease; [5] lower body mass index; [10] malnutrition; [5, 7, 8, 10, 11] inflammation; [5, 9–14] deficiencies of iron, [7, 8, 10, 14, 15] vitamin B12, [16] folate [17] or vitamin D; [18] hyperphosphatemia; [19] hyperparathyroidism; [13, 15] elevated levels of serum alkaline phosphatase; [15] inadequate dialysis; [8] infection; [7, 20, 21] malignancy; [7] use of ACE inhibitors or angiotensin receptor blockers; [13, 22] presence of a failed kidney transplant; [23] and anti-erythropoietin antibodies [24]. However, after excluding these conditions, a significant proportion of patients exhibit primary ESA-resistant anemia. The incidence and factors responsible for *primary* resistance to ESA are unknown.

ESA treatment targeting hemoglobin levels above 130 g/L in people with CKD is associated with deleterious [25] or neutral [26] impacts on survival and increased risks of stroke, vascular access thrombosis and hypertension without any reduction in cardiovascular events [25, 26]. However, recently published studies have demonstrated that poor response to ESA treatment, rather than achieved high hemoglobin, is associated with the observed adverse outcomes in CKD [2, 14, 27–30]. Unfortunately, there are no established therapies for primary ESA-resistant anemia [31]. The Handling Erythropoietin Resistance with Oxpentifylline (HERO) trial evaluated the effect of pentoxifylline on erythropoiesis resistance index (ERI) in patients with advanced CKD and primary ESA-hyporesponsive anemia [32]. We conducted a post-hoc analysis of the HERO Study to evaluate the determinants of severity of ESA resistance.

## Methods

Details of the HERO Study protocol and population are described elsewhere [32, 33]. In brief, the HERO Study (registration number Australian New Zealand Clinical Trials Registry 12608000199314) was a multi-centre, double-blind, randomized placebo-controlled trial to study the effect of pentoxifylline on ERI. The study was approved by ethics committees at all participating centres. All patients provided written informed consent prior to trial participation and the trial was conducted in accordance with the principles of the International Conference on Harmonisation Good Clinical Practice Guideline.

Between June 2009 and December 2011, the study enrolled 53 adult patients with stages 4 or 5 CKD (receiving dialysis treatment or estimated GFR <30 ml/min/1.73 m^2^) and ESA- resistant anemia on a stable dose of either erythropoietin or darbepoetin for at least 8 weeks. ESA-resistant anemia was defined as hemoglobin concentration ≤120 g/L and ERI ≥1.0 IU/kg/week per g/L for erythropoietin and ≥0.005 μg/kg/week per g/L for darbepoetin. ERI was calculated as weight-adjusted weekly dose of ESA divided by hemoglobin concentration, (expressed as IU/kg/week per g/L). ERI for darbepoetin-treated patients was converted to an erythropoietin-equivalent value using a dose conversion factor of 200:1.

Patients with an identifiable cause for their ESA hyporesponsiveness (such as iron deficiency, bleeding, inadequate dialysis, parathyroid hormone >100 pmol/L, malignancy or hematologic disorder, major surgery, infection, acute myocardial infarction or malignancy within the last 3 months) were excluded. Participants were randomized in a 1:1 ratio to receive pentoxifylline (Trental®, Sanofi-Aventis, Sydney, Australia) 400 mg daily orally or an identical matching placebo. The randomization was performed by an adaptive allocation algorithm designed to minimize imbalance in treatment groups across three variables: study site; CKD stage (4 or 5) and ESA class (erythropoietin or darbepoetin) using a password-protected web-based system. The follow up period was 4 months, unless a participant experienced a hemoglobin concentration <65 g/L or required a blood transfusion. The primary efficacy outcome was ERI. Secondary outcome variables were hemoglobin concentration, ESA dose, rate of blood transfusions, adverse events, quality of life and cost-effectiveness analysis. Of the 53 participants, plasma samples for four oxidative stress biomarkers (total F2-isoprostanes, protein carbonyls, glutathione peroxidase [GPX] and superoxide dismutase [SOD] activities) were available in 41 participants.

### Statistical analysis

This post-hoc analysis included only the baseline data from the main HERO Study and oxidative stress substudy. Results were expressed as frequencies (percentages) for categorical variables, mean ± standard deviation for continuous normally distributed variables and median [interquartile range] for continuous non-normally distributed variables. Participants were divided into tertiles of ERI (low, medium and high ERI). Differences between groups of patients were analysed by *χ*^2^ test for categorical data; one-way analysis of variance for continuous variables if data were normally distributed and Kruskal–Wallis test for non-normally distributed data. Simple linear regression was used to analyze the association between ERI and other variables. Non-normally distributed variables were appropriately transformed to improve normality of distribution. Associations between ERI and the following variables were analysed in linear regression models: gender, ethnicity, diabetes mellitus, cause of kidney disease, smoking status, ischemic heart disease, congestive heart failure, and body mass index category, age, reticulocyte count, total white cell count, ferritin, transferrin saturation, albumin, alkaline phosphatase, gamma-glutamyltransferase, alanine transaminase, aspartate transaminase, lactate dehydrogenase, albumin-corrected calcium, phosphate, parathyroid hormone, C-reactive protein, total F2-isoprostanes, protein carbonyls, GPX and SOD activities. Predictors of high ERI tertile versus low and medium ERI tertiles were determined by univariate multinomial logistic regression models. The same above mentioned variables were used in these models. Analysis was conducted using Stata/SE (version 11.2, Stata Corp., College Station, TX, USA).

## Results

### Patient characteristics

Baseline characteristics of the study population according to ERI tertiles are described in Table 1. The mean ERI values in the low, medium and high ERI tertiles were 1.4 ± 0.3, 2.3 ± 0.2 and 3.5 ± 0.8 IU/kg/week/gHb, respectively. Increasing ERI was associated with both higher ESA dose and lower hemoglobin level (Table 1). Serum alkaline phosphatase concentrations also increased with increasing ERI. Median [IQR] serum alkaline phosphatase levels in the low, medium and high ERI tertiles were 89 [64, 121], 99 [76, 134] and 148 [87, 175] U/L, respectively (*P* = 0.054, Table 1). There were no statistically significant differences observed between the ERI tertiles with respect to age, gender, ethnic origin, cause of kidney disease, smoking status, dialysis modality, comorbidities, body mass index, other laboratory values or oxidative stress markers (Table 1).

**Table 1:** Baseline characteristics according to tertiles of ERI

Variable	ERI tertile			*P*
	Low	Medium	High	
n	18	18	17	
ERI (IU/kg/wk/gHb)	1.4 ± 0.3	2.3 ± 0.2	3.5 ± 0.8	<0.001
ESA dose (IU/kg/wk)^a^	153 ± 37	233 ± 25	362 ± 79	<0.001
*Type of ESA*				0.8
Erythropoietin	11 (61 %)	12 (67 %)	12 (71 %)	
Darbepoetin	7 (39 %)	6 (33 %)	5 (29 %)	
Hemoglobin (g/L)	111 ± 8	102 ± 13	105 ± 10	0.05
Men	10 (56 %)	8 (44 %)	6 (35 %)	0.5
Age (years)	67.7 ± 13	58.4 ± 17.6	60.5 ± 15.5	0.2
*Ethnicity*				0.4
Caucasian	14 (78 %)	14 (78 %)	15 (88 %)	
Aboriginal or Torres Strait Islander	0	1 (6 %)	0	
Maori/Pacific Islander	0	1 (6 %)	0	
Asian	3 (17 %)	2 (11 %)	0	
Other	1 (6 %)	0	2 (12 %)	
*Cause of kidney disease* ^b^				0.9
Diabetes	8 (44 %)	8 (47 %)	5 (29 %)	
Glomerulonephritis	2 (11 %)	1 (6 %)	4 (24 %)	
Analgesic nephropathy	0	1 (6 %)	0	
Polycystic kidney disease	1 (6 %)	1 (6 %)	1 (6 %)	
Reflux nephropathy	1 (6 %)	1 (6 %)	1 (6 %)	
Renovascular disease	0	1 (6 %)	1 (6 %)	
Others	6 (33)	4 (24 %)	5 (29 %)	
*Type of dialysis* ^b^				0.4
Hemodialysis	17 (94 %)	16 (94 %)	17 (100 %)	
Peritoneal dialysis	1 (6 %)	0	0	
Pre-dialysis	0	1 (6 %)	0	
Diabetes mellitus^c^	10 (67 %)	9 (82 %)	6 (46 %)	0.2
Ischemic heart disease^c^	7 (47 %)	4 (36 %)	7 (54 %)	0.7
Congestive heart failure^c^	2 (13 %)	1 (9 %)	5 (38 %)	0.1
Cerebrovascular disease^c^	1 (7 %)	0	3 (23 %)	0.2
Peripheral vascular disease^c^	2 (13 %)	4 (36 %)	4 (31 %)	0.4
*Smoking status* ^b^				0.3
Never	9 (50 %)	5 (29 %)	9 (53 %)	
Former	8 (44 %)	11 (65 %)	5 (29 %)	
Current	1 (6 %)	1 (6 %)	3 (18 %)	
Body mass index (kg/m^2^)	28.6 ± 4.8	30.4 ± 7.4	29.2 ± 7.6	0.7
*Body mass index category*				0.3
Underweight (<18.5 kg/m^2^)	0	0	0	
Healthy weight (18.5 – 24.9 kg/m^2^)	5 (28 %)	4 (22 %)	6 (35 %)	
Overweight (25 – 29.9 kg/m^2^)	9 (50 %)	6 (33 %)	3 (18 %)	
Obese (>30 kg/m^2^)	4 (22 %)	8 (44 %)	8 (47 %)	
Hematocrit (%)	34 ± 3	32 ± 5	32 ± 3	0.7
Mean cellular volume (fL)	94.4 ± 7.8	95.3 ± 6.6	94.3 ± 5.9	0.9
Reticulocyte count (10^9^/L)	57 ± 21	74 ± 38	64 ± 31	0.3
Total white cell count (x 10^9^ per L)	6.8 ± 2	6.4 ± 2.1	6.6 ± 1.5	0.9
Platelet count (x 10^9^ per L)^d^	207 [164, 286]	188 [167, 207]	197 [164, 274]	0.8
Ferritin (μg/L)^d^	413 [241, 958]	527 [298, 647]	487 [315, 594]	0.9
Transferrin saturation (%)^d^	26 [20, 30]	22 [17, 30]	23 [22, 29]	0.6
Serum bicarbonate (mmol/L)	23.3 ± 4	22.6 ± 3	23.6 ± 3	0.6
Albumin (g/L)	36 ± 4	35 ± 4	35 ± 5	0.7
Serum alkaline phosphatase (U/L)^d^	89 [64, 121]	99 [76, 134]	148 [87, 175]	0.054
Gamma-glutamyltransferase (U/L)^d^	23 [16, 40]	25 [17, 60]	32 [21, 57]	0.4
Alanine transaminase (U/L)^d^	16 [11, 19]	15 [9, 22]	10 [8, 19]	0.2
Aspartate transaminase (U/L)^d^	18 [13, 22]	17 [12, 23]	14 [10, 17]	0.3
Lactate dehydrogenase (U/L)	224 ± 57	224 ± 85	265 ± 63	0.2
Albumin-corrected calcium (mmol/L)	2.3 ± 0.2	2.3 ± 0.1	2.2 ± 0.2	0.2
Phosphate (mmol/L)^e^	1.7 ± 0.5	1.7 ± 0.5	1.7 ± 0.5	0.9
Parathyroid hormone (pmol/L)^d,f^	17 [9, 35]	33 [17, 42]	32 [16, 65]	0.2
C-reactive protein (mg/L)	3 [2, 21]	12 [4, 28]	15 [7, 31]	0.06
*Oxidative stress substudy*				
n	14	13	14	
Total F2-isoprostanes (pg/mL)^d^	133 [87, 213]	143 [128, 200]	112 [67, 173]	0.3
Glutathione peroxidise activity (U/L)	177 (13)	181 (10)	174 (14)	0.4
Superoxide dismutase activity (U/mL)^d,f^	1.97 [1.41, 2.6]	2.01 [1.32, 2.44]	2.22 [1.71, 2.71]	0.7
Protein carbonyls (nmol/mg)^d^	0.56 [0.46, 0.6]	0.55 [0.46, 0.74]	0.55 [0.51, 0.69]	0.9

^a^Patients on darbepoetin were converted to an erythropoietin-equivalent dose using a conversion factor of 200:1

^b^1 missing data from medium ERI tertile

^c^Missing data- 3, 7 and 4 values missing from low, medium and high ERI tertiles, respectively

^d^Median [IQR]

^e^to convert phosphate level from mmol/L to mg/dL multiply by 3.1

^f^to convert parathyroid level from pmol/L to pg/mL multiply by 9.4

### Determinants of ESA resistance index

**Fig. 1:**
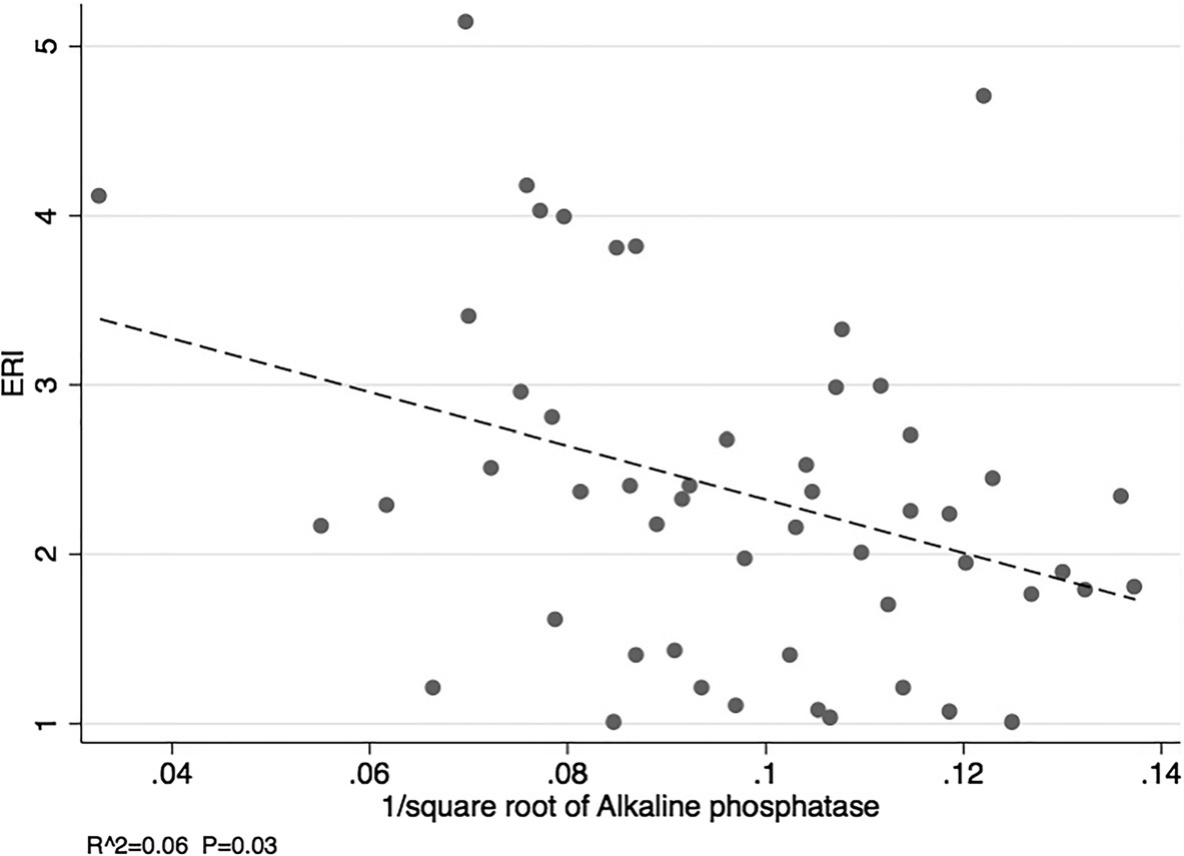
Scatterplot showing the association between serum alkaline phosphatase and ESA resistance index

Using simple linear regression, there was a weak but statistically significant association between ERI and alkaline phosphatase (R^2^ = 0.06, *P* = 0.03) (Fig. 1). On multinomial logistic regression, the risk of being in the high ERI tertile relative to the low ERI tertile increased with increasing alkaline phosphatase levels (*P* = 0.02). No other variables were significantly associated with ERI on univariate analysis (Tables 2 and 3).

**Table 2:** Associations between participant characteristics and ERI by simple linear regression

Variable	R^2^	*P* value
Women (Reference men)	−0.02	0.8
Non-Caucasians (Reference Caucasians)	−0.02	0.7
Diabetes mellitus (Reference non-diabetics)	0.01	0.2
Cause of kidney disease	−0.03	0.6
Smoking status	−0.02	0.6
Body mass index	0.02	0.2
Ischemic heart disease	−0.01	0.4
Congestive heart failure	0.02	0.2
Age	0.01	0.2
Reticulocyte count	−0.01	0.6
Total white cell count	−0.02	0.8
Ferritin^a^	−0.01	0.5
Transferrin saturation^b^	−0.01	0.6
Albumin	0.001	0.3
Alkaline phosphatase^c^	0.06	0.03
Gamma-glutamyltransferase^b^	0.01	0.2
Alanine transaminase^c^	0.02	0.1
Aspartate transaminase^c^	0.04	0.09
Lactate dehydrogenase^b^	0.07	0.05
Albumin-corrected calcium	0.05	0.06
Phosphate	−0.02	0.9
Parathyroid hormone^a^	0.04	0.09
C-reactive protein	0.03	0.09
Total F2-isoprostanes^b^	−0.01	0.5
Glutathione peroxidise activity^d^	−0.004	0.9
Superoxide dismutase activity^e^	−0.01	0.5
Protein carbonyls^e^	−0.03	0.9

The following non-normally distributed variables were transformed to normality of distribution:

^a^square root

^b^log-transformed

^c^reciprocal of square root

^d^power of 3

^e^reciprocal

**Table 3:** Associations between participant characteristics and ERI by univariate multinomial logistic regression

Variable	Coefficient	95 % confidence intervals	*P* value
Women (Reference men)	−0.83	−2.19 to 0.53	0.2
Non-Caucasians (Reference Caucasians)	0.76	−1.08 to 2.61	0.4
Diabetes mellitus (Reference non-diabetics)	0.85	−0.68 to 2.38	0.3
*Cause of kidney disease*			
Diabetes	Reference		
Glomerulonephritis	−1.16	−3.19 to 0.87	0.3
Analgesic nephropathy	NR	NR	NR
Polycystic kidney disease	−0.47	−3.46 to 2.52	0.8
Reflux nephropathy	−0.47	−3.46 to 2.52	0.8
Renovascular disease	NR	NR	NR
Others	−0.29	−1.92 to 1.34	0.7
*Smoking status*			
Never	Reference		
Former	0.47	−0.98 to 1.92	0.5
Current	−1.10	−3.54 to 1.35	0.4
*Body mass index category*			
Healthy weight	Reference		
Overweight	1.28	−0.48 to 3.05	0.2
Obese	−0.51	−2.20 to 1.18	0.6
Ischemic heart disease	−0.28	−1.78 to 1.20	0.7
Congestive heart failure	−1.40	−3.26 to 0.46	0.1
Age	0.03	−0.01 to 0.08	0.2
Reticulocyte count	−0.01	−0.03 to 0.02	0.5
Total white cell count	0.03	−0.33 to 0.40	0.9
Ferritin^a^	0.01	−0.09 to 0.11	0.8
Transferrin saturation^b^	0.05	−2.29 to 2.39	0.9
Albumin	0.06	−0.09 to 0.21	0.4
Alkaline phosphatase^c^	44.01	7.63 to 80.40	0.02
Gamma-glutamyltransferase^b^	−0.65	−1.60 to 0.30	0.2
Alanine transaminase^c^	−7.42	−16.80 to 1.96	0.1
Aspartate transaminase^c^	−8.50	−20.62 to 3.62	0.2
Lactate dehydrogenase^b^	−2.18	−5.06 to 0.69	0.1
Albumin-corrected calcium	3.46	−0.60 to 7.51	0.09
Phosphate	−0.01	−1.31 to 1.29	0.9
Parathyroid hormone^a^	−0.25	−0.55 to 0.05	0.1
C-reactive protein	−0.03	−0.08 to 0.01	0.2
Total F2-isoprostanes^b^	0.36	−0.69 to 1.42	0.5
Glutathione peroxidise activity^d^	NR	NR	NR
Superoxide dismutase activity^e^	1.49	−1.91 to 4.90	0.4
Protein carbonyls^e^	0.24	−1.26 to 1.74	0.8

*NR* Estimates could not be obtained due to small number of patients

The following non-normally distributed variables were transformed to normality of distribution:

^a^square root

^b^log-transformed

^c^reciprocal of square root

^d^power of 3

^e^ reciprocal

## Discussion

This secondary analysis of the HERO Study showed that serum alkaline phosphatase was associated with severity of ESA resistance in a selected group of patients with advanced CKD who did not have any identifiable cause of ESA-resistant anemia. No other factors were found to be associated with severity of ESA resistance.

In a study involving 38,328 ESKD patients receiving hemodialysis, Kalantar-Zadeh and colleagues reported a positive association between serum alkaline phosphatase level and ESA hyporesponsiveness [15]. Importantly, this association persisted even after adjusting for other known causes of anemia, such as older age, gender, diabetes mellitus, body mass index, iron studies, markers of bone disease, parathyroid level and markers of malnutrition. Other investigators have reported improvement in hemoglobin concentration and reductions in the serum alkaline phosphatase level and ESA dose after parathyroidectomy [34, 35]. Previous studies have also demonstrated that alkaline phosphatase is associated with mortality in ESKD patients receiving dialysis [36–39] and pre-dialysis patients with CKD [40–43]. A major difference between the present study and previous investigations is that the HERO study excluded patients with any identifiable cause of ESA-resistant anemia, such as deficiencies of iron or vitamin B12 or folate, bleeding, inadequate dialysis, severe hyperparathyroidism (PTH >100 pmol/L), malignancy or hematologic disorder, major surgery, infection, acute myocardial infarction or malignancy within the last 3 months. Indeed, this is the first study describing the determinants of severity of ESA resistance in patients with primary ESA-resistance, as previous studies included patients with no ESA resistance as well as those with known secondary causes of ESA resistance.

Approximately 31–37 % of ESKD patients receiving dialysis have raised levels of serum alkaline phosphatase [36, 44]. Serum alkaline phosphatase in the dialysis population is strongly associated with serum concentrations of parathyroid hormone and aspartate transaminase [36]. Although bone and liver alkaline phosphatase are found in equal proportions in healthy adults, 28 % of ESKD patients on haemodialysis with increased bone alkaline phosphatase have normal alkaline phosphatase levels [44]. In a study involving 800 ESKD patients receiving haemodialysis, Drechsler and colleagues showed a strong association between bone alkaline phosphatase and all-cause and cardiovascular mortality [37].

The most likely reason for the observed association between alkaline phosphatase and severity of ESA resistance in the present study is increased bone turnover and marrow fibrosis, since the median serum PTH levels in the middle and high ERI tertiles were 33 and 32 pmol/L, respectively compared with 17 pmol/L in the low ERI tertile [45]. In the current study, there was no statistically significant association observed between parathyroid hormone and primary ESA-resistance. It is important to note that the HERO Study excluded patients with a serum parathyroid hormone level greater than 100 pmol/L, such that included patients only had mild-to-moderate secondary hyperparathyroidism. Nevertheless, even at these relatively modest elevations of serum PTH, alkaline phosphatase was still significantly associated with ESA resistance.

A strength of the study was that it involved patients from multiple centers across two countries, enhancing the internal and external validity of the findings. On the other hand, the study was limited by a relatively small sample size, such that it is possible that some associations with severity of ESA resistance were not able to be ascertained due to a type 2 statistical error. Moreover, as multiple variables were evaluated in this study, the observed association between alkaline phosphatase and severity of ESA resistance could have been due to a type 1 statistical error. Bone-specific alkaline phosphatase, other markers of bone turnover and bone biopsies were not evaluated, thereby limiting more detailed exploration of the potential mechanisms underpinning the association between serum alkaline phosphatase and severity of ESA resistance. Since the HERO study included a highly selected group of patients with no identifiable cause of ESA-resistant anemia, the findings of this study may not be generalizable to patients with a known cause of ESA hyporesponsiveness. This finding is hypothesis generating and needs to be confirmed by other studies.

## Conclusions

Alkaline phosphatase was associated with severity of ESA resistance in patients with advanced CKD and no apparent secondary cause of ESA-resistance. Larger prospective studies are required to confirm this association.

## Abbreviations

ACE: Angiotensin converting enzyme
CKD: Chronic kidney disease
ERI: Erythropoiesis resistance index
ESA: Erythropoiesis stimulating agents
ESKD: End-stage kidney disease
GPX: Glutathione peroxidase
HERO: The Handling Erythropoietin Resistance with Oxpentifylline trial
IQR: Interquartile range
PTH: Parathyroid hormone
SOD: Superoxide dismutase
